# The effects of high HIV prevalence on orphanhood and living arrangements of children in Malawi, Tanzania, and South Africa

**DOI:** 10.1080/00324720701524292

**Published:** 2007-11-02

**Authors:** Victoria Hosegood, Sian Floyd, Milly Marston, Caterina Hill, Nuala McGrath, Raphael Isingo, Amelia Crampin, Basia Zaba

**Affiliations:** 1 London School of Hygiene and Tropical Medicine, Tanzania; 2 Karonga Prevention Study, Tanzania; 3 Africa Centre for Health and Population Studies, Tanzania; 4 National Institute for Medical Research, Tanzania

**Keywords:** HIV, AIDS, orphans, living arrangements, longitudinal studies, sub-Saharan Africa

## Abstract

Using longitudinal data from three demographic surveillance systems (DSS) and a retrospective cohort study, we estimate levels and trends in the prevalence and incidence of orphanhood in South Africa, Tanzania, and Malawi in the period 1988–2004. The prevalence of maternal, paternal, and double orphans rose in all three populations. In South Africa—where the HIV epidemic started later, has been very severe, and has not yet stabilized—the incidence of orphanhood among children is double that of the other populations. The living arrangements of children vary considerably between the populations, particularly in relation to fathers. Patterns of marriage, migration, and adult mortality influence the living and care arrangements of orphans and non-orphans. DSS data provide new insights into the impact of adult mortality on children, challenging several widely held assumptions. For example, we find no evidence that the prevalence of child-headed households is significant or has increased in the three study areas.

## Introduction

Most comparative studies of orphanhood and children's living arrangements in Africa have used data from cross-sectional surveys, including the Demographic and Health Surveys (DHS) and the UNICEF Multiple Indicator Cluster Surveys (MICS) (Bicego et al. 2003; Grassly et al. 2004; Monasch and Boerma 2004). Longitudinal community-based studies, including demographic surveillance systems (DSS), provide a complementary and under-utilized source of data on these topics; for example, they permit estimation of the incidence of orphanhood in addition to trends in its prevalence. A UNICEF-sponsored initiative encouraged investigators at several DSS sites to analyse and compare their orphanhood data (Floyd et al. 2005; Zaba et al. 2005). The data described in this paper were obtained from DSSs in Kisesa in Tanzania, Hlabisa in South Africa, and Karonga in Malawi, and two total population surveys and a retrospective cohort study in Karonga. We present estimates of the levels and patterns of the prevalence and incidence of orphanhood, and describe the living and care arrangements of orphans and non-orphans. We also discuss methodological issues that arise in the analysis and interpretation of longitudinal data on orphanhood and living arrangements.

## Background and methods

This section describes HIV prevalence and the emergence of the epidemic in the three study areas, and compares other demographic, social, and cultural characteristics influencing the living arrangements of children and households.

### Experience of HIV and AIDS

The HIV epidemics in Malawi and Tanzania began earlier than in South Africa and, in a reference to the recent flattening out of HIV prevalence, have been described as ‘mature’ epidemics (Crampin et al. 2003; Ghys et al. 2004; Walker et al. 2004). Population-based HIV data are available from all three study areas. In Karonga, HIV prevalence in adults was 0.1 per cent in the early 1980s, 2 per cent in the late 1980s, and around 13 per cent in the late 1990s (Crampin et al. 2003). In Kisesa, the serosurvey prevalence data (adjusted for age and sex) in adults aged 15–44 years was 6 per cent in 1994, 7 per cent in 1997, and 8 per cent in 2004 (Boerma et al. 1999a; Mitwa et al. 2006). Crude antenatal HIV seroprevalence in Hlabisa increased from 4.2 per cent (95 per cent CI, 3.0–5.7) in 1992, to 14 per cent (95 per cent CI, 10.4–18.4) in 1995 (Coleman and Wilkinson 1997), and to 41 per cent (95 per cent CI, 34.7–47.9) by 1998 in the largest clinic (Wilkinson et al. 1999). According to population-based data from the Hlabisa-DSS, HIV prevalence in 2003–4 was 22 per cent for women aged 15–49 years and for men aged 15–54 years (Welz et al. in press).

AIDS is currently the leading cause of adult death in all study areas according to estimates based on verbal autopsies. The Karonga-DSS found that 60 per cent of men and 66 per cent of women aged 15–44 years who died in the period 2003–5 died from AIDS (Jahn et al. 2005). According to the Hlabisa-DSS, 48 per cent of all deaths of adults aged 15 years and older were due to AIDS in 2000 (Hosegood et al. 2004b). Similarly, the Kisesa-DSS found that AIDS accounted for 50 per cent of all adult deaths in 1994–96 (Boerma et al. 1999b).

### Marriage and migration

Patterns of marriage, cohabitation of parents, and adult migration—important influences on children's living arrangements—differ between the study areas. Karonga has a higher rate of marriage than Kisesa, and rates in both are markedly higher than those in Hlabisa. The Karonga-DSS found that 81 per cent of women and 55 per cent of men aged 15–49 years were married in 2002 (Jahn, personal communication). In contrast, marriage rates are very low in South Africa, having declined substantially since the 1980s (Hunter 2002, 2007; Denis and Nstmane 2006). The Hlabisa-DSS found that in 2001 25 per cent of women and 20 per cent of men aged 18–59 years were married (Hosegood and Preston-Whyte 2002). In Karonga, virtually all marital couples cohabit, while in Kisesa and Hlabisa, higher labour migration and union instability mean that more than one in ten and one in three couples, respectively, live apart (Boerma et al. 2002; Floyd et al. 2006; Hosegood et al. 2006; Kishamawe et al. 2006).

Adult mobility is high in all three areas but is a defining characteristic of the Kisesa and Hlabisa populations. The Kisesa-DSS showed that, over the period 1994–98, approximately 11 per cent of adults aged 15–59 years migrated per year (Urassa et al. 2001; Boerma et al. 2002). In Hlabisa, of those in the most mobile age group of 25–29 years, 40 per cent of men and 30 per cent of women who formerly lived in the area maintain links with a household there through visits and remittances (Hosegood and Timæus 2005) but are normally resident elsewhere. In Malawi and Tanzania the high rates of migration among young females are mostly the result of marriage rather than labour migration as is the case in South Africa. Children too are mobile in all the study areas and may move with or independently of adults (Chirwa et al. 2005; Ford and Hosegood 2005).

### Data sources

Detailed descriptions of the study areas and methods are presented elsewhere (Urassa et al. 2001; Boerma et al. 2002; Crampin et al. 2002; Hosegood and Timæus 2005). For clarity we attach place names to the data sources; these may differ from the names given in previously published papers.

#### Hlabisa-DSS

The Africa Centre Demographic Information System (ACDIS) is located in the Umkhanyakude district in northern KwaZulu-Natal, South Africa. Started in 2000, ACDIS has a surveillance population that comprises approximately 89,000 members in 11,000 households. Household updates are conducted twice a year (Hosegood et al. 2005; Hosegood and Timæus 2005).

#### Karonga-DSS, Karonga-surveys, and Karonga-cohort

The Karonga Prevention Study (KPS) has conducted several community-based studies in the Karonga district of northern Malawi. Two population surveys were conducted in the 1980s (Chirwa et al. 2005), and a retrospective cohort study (1998–2000) collected data on 197 HIV-positive index adults and 396 matched HIV-negative adults and their families (Crampin et al. 2002). In 2002, the Continuous Registration System (Karonga-DSS) started. Informants provide monthly demographic updates on 5,800 households with 30,000 members. A household census is repeated every 2 years (Jahn et al. 2007).

#### Kisesa-DSS

The Kisesa Open Cohort is a DSS located in the Magu district in the Mwanza region in north-west Tanzania. Half-yearly demographic surveillance updates were started in 1994 for a population of approximately 20,000 people living in 4,600 households (Boerma et al. 2002), which had risen to 27,000 people in 4,700 households by 2004 (Zaba, personal communication).

### Methodological issues

Several methodological issues need to be considered in the analysis and interpretation of longitudinal data on orphans.

#### (i) Identifying parental survival status

In these three DSSs, the survival status of a child's parent can be established in two ways. First, it can be established through routine prospective observations of the child and its linked parent when both are registered individuals in the DSS. Many children are linked to their parents from the time they are registered as new births in the surveillance area. In the Kisesa-DSS, 78 per cent of all children under 18 years of age can be linked to their mothers and 56 per cent to their fathers. In the Hlabisa-DSS, the proportions linked are 78 and 42 per cent, respectively, and in the Karonga-DSS, 92 and 81 per cent. The second way of linking parent and child uses data from a direct question about whether a child's parent is alive. This ‘orphanhood’ question was asked of every child in the Kisesa-DSS during the baseline census in 1994, and again in the 2000 and 2004 rounds. In the Hlabisa-DSS orphanhood data are collected directly when a child is registered and (since 2004) at every household update visit. On the other hand, the Karonga-DSS collected orphanhood data directly in the baseline census and subsequently when new children were registered.

#### (ii) Defining orphans

There is a variety of definitions of maternal, paternal, and double orphanhood. We use the UNAIDS definitions: maternal orphans are children whose mothers have died and whose father is alive or whose survival status is unknown, while paternal orphans have the opposite characteristics. Double orphans are those who have lost both parents (Grassly and Timæus 2005).

#### (iii) Age limits

In Malawi and Tanzania, relatively few children enrol in secondary school and the median age of marriage for women is around 18 years. In contrast, the majority of children in South Africa will still be in full-time education at age 18 years, and 18 is the minimum legal age of marriage. In order to avoid distorting comparisons of children's living arrangements by including people who started or joined other households following marriage, an upper age limit of under 15 years is used for the Kisesa-DSS and the Karonga-DSS and under 18 years for the Hlabisa-DSS (Table 3).

#### (iv) Defining households

The criteria used to define a household in each study area are important when comparing data on living arrangements. While in all areas investigators rely on respondents to identify the members of their household, only resident household members are eligible for registration and follow-up in the Karonga-DSS and Kisesa-DSS (Urassa et al. 2001; Jahn et al. 2007). Follow-up stops when a household member migrates to live outside the surveillance area. Owing to the high level of circular migration in rural South Africa, in the Hlabisa-DSS both resident and non-resident household members are registered and followed up (Hosegood et al. 2004a). The survival status of resident and non-resident parents is updated in the same way. Thus, children and parents who are coresident can be distinguished from children and parents who belong to the same household but live apart. For the purposes of comparability, the orphanhood estimates for Hlabisa-DSS comprise resident children only (Table 3). The data used for descriptions of the living arrangements of children in Hlabisa and Karonga (Table 3) include data on resident and non-resident household heads.

## Results

### (i) Orphanhood levels and trends

Table 1 presents two estimates of annual orphanhood for each study area for children under 18 years of age and children under 15 years of age, as well as orphanhood estimates reported by the most recent DHS conducted in Malawi, Tanzania, and South Africa. In each DSS site, the prevalence of each category of orphanhood increased between the early and later periods.

**Table 1:** Percentage of children less than 18 years of age who are orphans, by age group and orphanhood type from DSSs, population surveys, and DHS surveys in Malawi, Tanzania, and South Africa, 1988–2004

Country, survey, and year	Age	Maternal orphan %	Paternal orphan %	Double orphan %
*South Africa*				
Hlabisa-DSS, 2004	0–4	2	4	<1
	5–9	7	12	2
	10–14	12	20	4
	15–17	14	25	5
	<15	7	11	2
	<18	7	13	2
Hlabisa-DSS, 2000	<15	3	8	1
	<18	3	9	1
DHS, 1998	<15	2	9	1
*Malawi*				
Karonga-DSS, 2003	0–4	<1	3	<1
	5–9	4	10	1
	10–14	8	18	3
	15–17	15	27	8
	<15	4	9	1
	<18	5	11	2
Karonga-surveys, 1988	<15	3	6	<1
	<18	4	7	<1
DHS, 2000	<15	5	8	2
*Tanzania*				
Kisesa-DSS, 2004	0–4	1	3	<1
	5–9	4	8	1
	10–14	9	14	3
	15–17	11	20	5
	<15	4	8	1
	<18	5	9	2
Kisesa-DSS, 1994	<15	3	6	<1
	<18	4	7	1
DHS, 1996	<15	3	6	1

By the end of the 1990s, the prevalence of maternal orphanhood in all study areas was similar (2–3 per cent among under-15s). In Karonga, the proportions of orphans of all types increased between the end of the 1980s and 2004, with the largest increase occurring in the proportion of paternal orphans. The proportion of maternal orphans found by the Hlabisa-DSS also doubled, but since the level of paternal orphanhood was already very high by 2000 the increase seemed less dramatic over the subsequent few years. The increase in orphanhood prevalence found in the Kisesa-DSS was lower than that found in the other areas, probably because of the lower HIV prevalence. In 1999, national HIV prevalence in Tanzania (8 per cent) was half that of Malawi in 2000 (16 per cent). The most recent estimates of paternal orphanhood are high in all study areas. In Kisesa and Hlabisa a quarter of all children in the age group 15–17 years lost a father. However, the level of double orphans in Hlabisa is not as high as in the other two areas, suggesting that high rates of non-AIDS mortality in men may account for much of this excess mortality among fathers. The national DHS estimates in the year closest to that for which the DSS estimates are made are similar to the DSS estimates.

### (ii) Orphanhood incidence

Orphanhood incidence can be calculated using the longitudinal DSS data. Incidence is calculated as the number of maternal deaths (or paternal deaths) per 1,000 person-years among children in each age group whose mother was living at the start of follow-up. For children whose parent died, the exposure period is calculated as the period between the start of follow-up and the date the parent died. For children who out-migrated, ceased to be a member of the household, or died, the years of follow-up are censored at the point that observations of the child stopped. Incidence rates and confidence intervals were calculated using Poisson regression. Table 2 shows the incidence rates derived from the Kisesa-DSS and the Hlabisa-DSS. In Hlabisa, where the HIV epidemic has not yet stabilized, the incidence of maternal orphanhood in children under 18 years of age (14.9 per 1,000 person-years) is considerably higher than in Kisesa (6.7 per 1,000 person-years). Paternal orphanhood incidence is also higher although the difference is not as large. Assuming incidence rates remain stable in the population covered by the Hlabisa-DSS, 24 per cent of surviving children are predicted to become maternal orphans by the age of 18 years, and 33 per cent to become paternal orphans.

**Table 2a:** Incidence of orphanhood per 1,000 person-years (95% CI) in the Kisesa-DSS, Tanzania (1994–2004)

	Paternal orphanhood		Maternal orphanhood	
		
Age in years	Person-years (1,000s)	Incidence (95% CI)	Person-years/1,000	Incidence (95% CI)
<1	4.1	7.8 (5.8–11.6)	5.9	2.7 (1.7–4.6)
1–4	22.9	8.2 (6.9–9.8)	30.9	3.7 (2.7–4.3)
5–9	22.7	10.6 (8.8–12.4)	29.4	7.2 (5.7–8.1)
10–14	17.9	15.4 (12.8–17.7)	21.6	10.0 (8.2–11.7)
15–17	8.4	15.3 (12.3–18.5)	10.0	10.3 (8.0–12.6)
0–17	75.1	11.4 (9.8–12.9)	97.9	6.7 (5.6–7.5)

**Table 2b:** Incidence of orphanhood per 1,000 person-years (95% CI) in the Hlabisa-DSS, South Africa (2000–2004)

	Paternal orphanhood		Maternal orphanhood	
		
Age in years	Person-years (1,000s)	Incidence (95% CI)	Person-years/1,000	Incidence (95% CI)
<1	8.3	8.2 (6.5–10.4)	8.7	7.1 (5.5–9.1)
1–4	34.5	14.6 (13.4–16.0)	36.4	9.7 (8.8–10.8)
5–9	44.4	19.2 (18.0–20.5)	47.8	15.0 (14.0–16.2)
10–14	41.8	22.3 (20.9–23.8)	46.7	18.8 (17.6–20.1)
15–17	22.8	24.9 (23.0–27.0)	26.4	17.4 (15.9–19.1)
0–17	151.8	19.3 (18.6–20.0)	166.3	14.9 (14.3–15.5)

### (iii) Parents' and children's living arrangements

There are marked differences between the three sites in the living arrangements of both non-orphans and orphans (Table 3). According to the Karongacohort and the Kisesa-DSS, the majority of non-orphans in these areas live with both parents—86 and 63 per cent, respectively, while only 27 per cent of non-orphans do so in Hlabisa according to its DSS. Indeed, in Hlabisa 22 per cent of non-orphans live with neither parent. In all three study areas, the majority of children whose mothers are alivelive with their mothers whatever the survival status of the father. The likelihood of maternal orphans living with the father varies. The Karonga-cohort yields the highest figure (68 per cent) and the Hlabisa-DSS the lowest (19 per cent).

**Table 3:** Living arrangements of children under 15 years of age: Karonga-cohort in 1998 (Malawi), Karonga-DSS in 2004 (Malawi), Kisesa-DSS in 2004 (Tanzania), and <18 years in Hlabisa-DSS in 2004 (South Africa)^1^

	Non-orphan				Paternal orphan				Maternal orphan				Double orphan			
				
	% Karonga-cohort	Karonga-DSS	% Kisesa-DSS	% Hlabisa-DSS	% Karonga-cohort	% Karonga-DSS	% Kisesa-DSS	% Hlabisa-DSS	% Karonga-cohort	% Karonga-DSS	% Kisesa-DSS	% Hlabisa-DSS	% Karonga-cohort	% Karonga-DSS	% Kisesa-DSS	% Hlabisa-DSS
*Co-residency with parents*																
Both parents	86	71	63	27	–	–	–	–	–	–	–	–	–	–	–	–
Mother only	6	15	20	47	77	68	53	70	–	–	–	–	–	–	–	–
Father only	5	4	4	4	–	–	–	–	68	41	25	19	–	–	–	–
Neither parent	3	10	13	22	23	32	43	30	32	59	75	81	100	100	100	100
*Household head's relationship to child*																
Father	91	79	66	38	–	–	–	–	61	50	34	21	–	–	–	–
Mother	1	2	6	4	40	46	45	46	–	–	–	–	–	–	–	–
Grandparent	4	17	22	42	30	43	30	28	18	38	40	47	29	69	50	44
Other relative	4	2	5	12	30	11	25	21	11	12	24	27	62	31	48	46
Non-relative	–	0	0.0	4	1	–	0	6	11	–	1.5	5	10	–	2	11
*Age of household head (years)*																
<15	0	0	0	–	0	0	0	–	0	0	0	<1	0	0	0	–
15–17	0	<1	0	–	0	0	1	–	0	0	0	<1	0	0	0	–
18–29	2	14	9	2	1	5	11	5	7	10	6	4	19	9	12	15
30–39	35	31	29	15	19	20	17	20	7	18	18	12	10	10	14	13
40–49	33	24	27	31	30	24	28	32	39	19	20	25	33	14	15	18
50–59	19	14	19	24	21	15	20	18	32	18	19	23	10	16	20	17
60+	10	16	16	28	30	36	23	24	14	35	36	38	29	51	39	37
*Sex of household head*																
Male	98	88	85	75	47	41	49	36	89	78	79	67	81	72	67	53
Female	2	12	15	25	53	59	51	64	11	22	21	33	19	28	33	47
Percentage living in a household with at least one woman 18–49	98	95	91	90	91	90	83	88	71	80	76	74	71	69	75	75

*N*	644	11,520	10,802	16,759	91	1,051	753	2,565	28	323	329	1,104	21	183	144	595

1 These estimates use data on only those children for whom the survival status of both parents is known.

The relationship of each member to the head of the household is the only variable that can be used to compare the patterns of intra-household relationships (Table 3). The Karonga-cohort and the Kisesa-DSS show that most non-orphans in these areas live in households headed by the non-orphan's father. In contrast, in South Africa a minority of non-orphans do so (38 per cent). The proportion living in the same household is higher than the proportion living with the father because some fathers will be non-resident household heads. In all sites, the majority of children who have lost a father live with the mother or grandparents. Orphans, especially paternal orphans, are more likely to live in female-headed households than are non-orphans. Significantly, in all three study areas, it is extremely rare for children, regardless of orphanhood status, to live in a household headed by someone under 18 years of age, that is, the type of household often described as a ‘*childheaded household*’.

## Discussion

The DSS data show increasing orphanhood prevalence in the three populations over the last decade. The prevalence of paternal orphanhood is substantially higher than that of maternal orphanhood in all populations, partly because fathers are on average older than mothers but also in part because of age and sex differentials in HIV infection and survival times. The orphanhood prevalence found by the three DSSs are consistent with findings based on DHS data reported in other comparative studies of orphanhood (Bicego et al. 2003; Grassly et al. 2004; Monasch and Boerma 2004). The Karonga-DSS and Hlabisa-DSS show very high rates of paternal mortality (approximately 25 per cent for 15–17 year olds). In Hlabisa this reflects high mortality among young adult men owing to AIDS, as well as a high proportion of violent and accidental deaths (Hosegood et al. 2004b; Timæus 2005). A useful feature of DSS data is that they allow estimation of the incidence of orphanhood, which is an indicator of future trends in its prevalence. The incidence of paternal orphanhood is higher than that of maternal orphanhood in Kisesa and Hlabisa-DSS, a finding that is the reverse of that predicted by earlier model-based estimates, which suggested that higher maternal than paternal orphanhood prevalence would be the common experience of countries most severely affected by AIDS (UNAIDS et al. 2004). In Hlabisa, where the HIV epidemic has not yet stabilized, orphanhood prevalence will continue to rise in the next decade. The estimates of its incidence derived from the Hlabisa-DSS resemble those based on a large Zimbabwean cohort study (1998–2003), which also showed similar levels of HIV (Watts et al. 2005).

The living arrangements of orphaned and non-orphaned children vary considerably between the three populations. In Karonga and Kisesa, the majority of non-orphans live with both parents, 86 and 66 per cent, respectively, a pattern that contrasts markedly with that found in Hlabisa, where only 27 per cent of non-orphans live with both parents. These differences cannot be attributed solely to labour migration. Variations in patterns of cohabitation, extra-marital fertility, and union stability are even more important than migration as influences on whether a child's parents live together. Like Monasch and Boerma (2004), we find that the pattern of parent–child co-residence among non-orphans is a good predictor of the pattern among orphans. In Karonga, if one parent dies, the surviving parent continues to live with the children in most cases; 77 per cent of paternal orphans live with the mother and 68 per cent of maternal orphans live with the father. In Kisesa and Hlabisa, on the other hand, while the majority of paternal orphans live with the mother, 58 and 70 per cent, respectively, only 30 and 19 per cent of maternal orphans, respectively, live with the father.

In Karonga and Kisesa the long-term responsibility for fostering older children is usually undertaken by an aunt, uncle, or older sibling. Children cared for by relatives will in most cases have joined a new household on being fostered. In Hlabisa, however, almost half of all non-orphans live in a household headed by their grandparent, a finding that reflects the well-established South African tradition of grandparents rearing their grandchildren whether or not the child's parents are alive (Preston-Whyte 1974, 1993).

We found no evidence in any of the populations that increases in orphanhood prevalence had led to a substantial increase in the number of child-headed households. In Karonga and Kisesa, no child-headed households were identified and fewer than ten were found in Hlabisa. This finding raises questions about the appropriateness of the prominence given to these households in HIV impact studies and programmes for children affected by HIV and AIDS (UNAIDS et al. 2004).

In the study of orphanhood, a DSS has several advantages over cross-sectional surveys. Repeated visits to households by fieldworkers can identify children living in them without their parents (such as children who work for the household) who might be overlooked in a single visit. The adoption effect, a potential source of bias in surveys, may also be minimized through repeated household visits and prospective record linkage (Timæus and Nunn 1997).

This was the first comparative family demography study to use data from several DSSs. By showing the problems of extracting comparable indicators of orphanhood and intra-household relationships, it has highlighted challenges that arise in analysing comparative data from these sources. While many DSSs collect information on living arrangements, each was designed independently to support different research agendas. Even direct comparisons of core demographic data are constrained by differences in variables, definitions, and coding. We hope that comparative exercises like the one presented here will promote the collection of data that could extend possibilities for comparative work. For example, it might encourage organizers of DSSs to identify biological fathers and to ask direct questions about parental survival.

The levels of and trends in orphanhood prevalence in these three study areas are worrying evidence of the impact that the HIV epidemic is having on the lives of children and families in Africa. The high incidence of orphanhood shows that without effective and accessible interventions to reduce parental mortality, the proportion of orphaned children will continue to rise. Government programmes to provide antiretroviral therapy have started in all three populations, but in the early years of these programmes they are unlikely to reduce adult mortality sufficiently to avoid further increases in the overall prevalence of orphanhood.

DSS data have been largely under-utilized in studies of social change and its impact on children in Africa. They are a potentially useful source of contextual information that can inform the design of programmes directed at children and their households, especially if they collect data on orphans and vulnerable children. While the generalizability of a single DSS's findings are inevitably constrained by the fact that its data are collected from people living in one small area, the potential for contributing to effective policy can be greatly improved if the findings are replicated in several areas. This point is well illustrated by the fact that we found scarcely any child-headed households in the three areas compared in our study.

## References

[b1] Bicego G., Rutstein S., Johnson K. (2003). Dimensions of the emerging orphan crisis in sub-Saharan Africa. Social Science and Medicine.

[b2] Boerma J. T., Urassa M., Senkoro K., Klokke A., Ng'weshemi J. (1999a). Spread of HIV infection in a rural area of Tanzania. AIDS.

[b3] Boerma J. T., Urassa M., Senkoro K., Klokke A., Zaba B., Ng'weshemi J. Z. L. (1999b). Levels and causes of adult mortality in rural Tanzania with special reference to HIV/AIDS. Health Transition Review.

[b4] Boerma J. T., Urassa M., Nnko S., Ng'weshemi J., Isingo R., Zaba B., Mwaluko G. (2002). Sociodemographic context of the AIDS epidemic in a rural area of Tanzania with a focus on people's mobility and marriage. Sexually Transmitted Infections.

[b5] Chirwa T., Ponnighaus J., Malema S., Kileta S., Zaba B., Floyd S., Bliss L., Fine P. E. M. (2005). Household dynamics in northern Malawi during the 1980s. Southern African Journal of Demography.

[b6] Coleman R. L., Wilkinson D. (1997). Increasing HIV prevalence in a rural district of South Africa from 1992 through 1995. Journal of Acquired Immune Deficiency Syndrome.

[b7] Crampin A. C., Floyd S., Glynn J. R., Sibande F., Mulawa D., Nyondo A., Broadbent P., Bliss L., Ngwira B., Fine P. E. M. (2002). Long-term follow-up of HIV-positive and HIV-negative individuals in rural Malawi. AIDS.

[b8] Crampin A. C., Glynn J. R., Ngwira B. M. M., Mwaungulu F. D., Ponnighaus J. M., Warndorff D. K., Fine P. E. M. (2003). Trends and measurement of HIV prevalence in northern Malawi. AIDS.

[b9] Denis P., Nstmane R., Morrell R., Richter L. (2006). Absent fathers: why do men not feature in stories of families affected by HIV/ AIDS in KwaZulu Natal?. Baba: Men and Fatherhood in South Africa.

[b10] Floyd S., Marston M., Hosegood V., Scholten F., Zaba B. (2005). UNICEF Project Report. HIV and Orphanhood: Final Report on Phase 3.

[b11] Floyd S., Crampin A. C., Glynn J. R., Madise N., Mwenebabu M., Mnkhondia S., Ngwira B., Zaba B., Fine P. E. M. (2006). The long-term social and economic impact of HIV on the spouse of infected individuals in northern Malawi.

[b12] Ford K., Hosegood V. (2005). AIDS mortality and the mobility of children in KwaZulu Natal, South Africa. Demography.

[b13] Ghys P. D., Brown T., Grassly N. C., Garnett G., Stanecki K. A., Stover J., Walker N. (2004). The UNAIDS Estimation and Projection Package: a software package to estimate and project national HIV epidemics. Sexually Transmitted Infections.

[b14] Grassly N. C., Lewis J. J. C., Mahy M., Walker N., Timæus I. M. (2004). Comparison of household-survey estimates with projections of mortality and orphanhood in sub-Saharan Africa in the era of HIV/AIDS. Population Studies.

[b15] Grassly N. C., Timæus I. M. (2005). Methods to estimate the number of orphans as a result of AIDS and other causes in sub-Saharan Africa. Journal of Acquired Immune Deficiency Syndrome.

[b16] Hosegood V., Preston-Whyte E. (2002). Marriage and partnership patterns in rural KwaZulu Natal, in Population Association of America Conference Abstracts, Atlanta, USA.

[b17] Hosegood V., McGrath N., Herbst K., Timæus I. M. (2004a). The impact of adult mortality on household dissolution and migration in rural South Africa. AIDS.

[b18] Hosegood V., Vanneste A.-M., Timæus I. M. (2004b). Levels and causes of adult mortality in rural South Africa. AIDS.

[b19] Hosegood V., Benzler J., Solarsh G. (2005). Population mobility and household dynamics in rural South Africa: implications for demographic and health research. Southern African Journal of Demography.

[b20] Hosegood V., Timæus I. M., van der Walle E. (2005). Household composition and dynamics in KwaZulu Natal, South Africa: mirroring social reality in longitudinal data collection. African Households. Census Data.

[b21] Hosegood V., Rochat T., Fitzgerald M., Barnighausen T., Adjei G., Baetzing-Feigenbaum J. (2006). Marriage and partnership patterns of HIV positive people in rural South Africa: implications for couple counselling in VTC and ART programmes.

[b22] Hunter M (2002). The materiality of everyday sex: thinking beyond ‘prostitution’. African Studies.

[b23] Hunter M (2007). The changing political economy of sex in South Africa: the significance of unemployment and inequalities to the scale of the AIDS pandemic. Social Science and Medicine.

[b24] Jahn A., Mwaungulu F. D., Msiska V., Crampin A., Kachiwanda L., Kasimba S., McGrath N., Fine P. E. M., Zaba B. (2005). What is the true death toll of AIDS in rural Malawi? Population estimates from the Karonga Continuous Registration System, in National AIDS Commission Annual Meeting Abstracts.

[b25] Jahn A., Crampin A. C., Glynn J. R., Mwinuka V., Mwaiyeghele E., Mwafilaso J., Mwaiyeghele E., Mwinuka V., Zaba B. (2007). Evaluation of a village-informant driven demographic surveillance system in Karonga, Northern Malawi. Demographic Research.

[b26] Kishamawe C., Vissers D. C., Urassa M., Isingo R., Mwaluko G., Borsboom G. J., Voeten H. A., Zaba B., Habbema J. D., de Vlas S. J. (2006). Mobility and HIV in Tanzanian couples: both mobile persons and their partners show increased risk. AIDS.

[b27] Mitwa W., Urassa M., Isingo R., Ndege M., Marston M., Zaba B., Changalucha J. (2006). HIV prevalence and incidence trends in a rural population in Northern Tanzania during 1994-2004.

[b28] Monasch R., Boerma J. T. (2004). Orphanhood and childcare patterns in sub-Saharan Africa: an analysis of national surveys from 40 countries. AIDS.

[b29] Preston-Whyte E, Hammond-Tooke W. (1974). Kinship and marriage. The Bantu-Speaking Peoples of Southern Africa.

[b30] Preston-Whyte E (1993). Women who are not married: fertility, ‘illegitimacy’, and the nature of households and domestic groups among single African women in Durban. South African Journal of Sociology.

[b31] Timæus I. M., Nunn A. J. (1997). Measurement of adult mortality in populations affected by AIDS: an assessment of the orphanhood method. Health Transition Review.

[b32] Timæus I. M, Carael M., Glynn J. (2005). Impact of HIV on mortality in Southern Africa: evidence from demographic surveillance. HIV, Resurgent Infections and Population Change in Africa.

[b33] UNAIDS, UNICEF, and USAID (2004). Children on the Brink.

[b34] Urassa M., Boerma J. T., Isingo R., Ngalula J., Ng'weshemi J., Mwaluko G., Zaba B. (2001). The impact of HIV/AIDS on mortality and household mobility in rural Tanzania. AIDS.

[b35] Walker N., Grassly N. C., Garnett G. P., Stanecki K. A., Ghys P. D. (2004). Estimating the global burden of HIV/AIDS: what do we really know about the HIV pandemic?. Lancet.

[b36] Watts H., Lopman B., Nyamukapa C., Gregson S. (2005). Rising incidence and prevalence of orphanhood in Manicaland, Zimbabwe, 1998 to 2003. AIDS.

[b37] Welz T., Hosegood V., Jaffar S., Batzing-Feigenbaum J., Herbst K., Newell M. (2007). Continued very high prevalence of HIV infection in rural KwaZulu-Natal, South Africa: a population-based longitudinal study. AIDS.

[b38] Wilkinson D., Connolly C., Rotchford K. (1999). Continued explosive rise in HIV prevalence among pregnant women in rural South Africa. AIDS.

[b39] Zaba B., Whitworth J., Marston M., Nakiyingi J., Ruberantwari A., Urassa M., Issingo R., Mwaluko G., Floyd S., Nyondo A., Crampin A. (2005). HIV and mortality of mothers and children. Evidence from cohort studies in Uganda, Tanzania, and Malawi. Epidemiology.

